# Increased production of viral proteins by a 3'-LTR-deleted infectious clone of human T-cell leukemia virus type 1

**DOI:** 10.1186/1743-422X-6-229

**Published:** 2009-12-24

**Authors:** Takeo Ohsugi

**Affiliations:** 1 Division of Microbiology and Genetics, Center for Animal Resources and Development, Institute of Resource Development and Analysis, Kumamoto University, 2-2-1 Honjo, Kumamoto 860-0811, Japan

## Abstract

We previously reported that a full-length provirus of HTLV-1 was directly constructed from the HTLV-1-transformed cell line MT-2 using overlapping polymerase chain reaction (PCR) and cloned into a plasmid vector (pFL-MT2). 293T cells transfected with pFL-MT2 alone did not produce virus particles because there was no expression of the viral transactivator protein Tax, whereas cells transfected with pFL-MT2 plus a Tax expression vector produced virus-like particles. In the process of constructing the HTLV-1 provirus by overlapping PCR, we also constructed an incomplete molecular clone, in which the 3' long terminal repeat (LTR) was replaced with the endogenous human gene, which resulted in the expression of a *tax *gene shorter by 43 bp. This incomplete molecular clone alone expressed Tax and produced the viral protein in transfected cells. Various clones were then constructed with different lengths of the 3' LTR and lacking the reverse-direction TATA box. The clones contained over 113 bp of the 3' LTR, with no reverse-direction TATA box, which might express the full-length *tax *gene, and did not produce the viral antigen. These results suggest that Tax in which the C-terminal portion is deleted is more strongly expressed than the wild-type protein and has transcriptional activity.

## Findings

Human T-cell leukemia virus type 1 (HTLV-1) was the first human retrovirus to be isolated [1,2]. It causes an aggressive malignancy known as adult T-cell leukemia/lymphoma, as well as inflammatory diseases such as HTLV-1-associated myelopathy/tropical spastic paraparesis, after a very prolonged period of latency, often lasting between 20 and 50 years [3,4]. The *tax *gene product encoded by the *pX *region of HTLV-1 appears to be a key element in the development of HTLV-1-associated diseases [5-7]. Tax enhances productive virus replication by driving gene transcription via the cAMP-responsive element located in the viral long terminal repeat (LTR) [8,9]. Tax also activates the expression of many cellular genes, including genes that encode cytokines, cytokine receptors, and immediate early transcription factors, via the activation of several cellular signal transduction pathways, such as the nuclear factor kappaB (NF-κB) and serum response factor (SRF) pathways [10-12].

**Figure 1:**
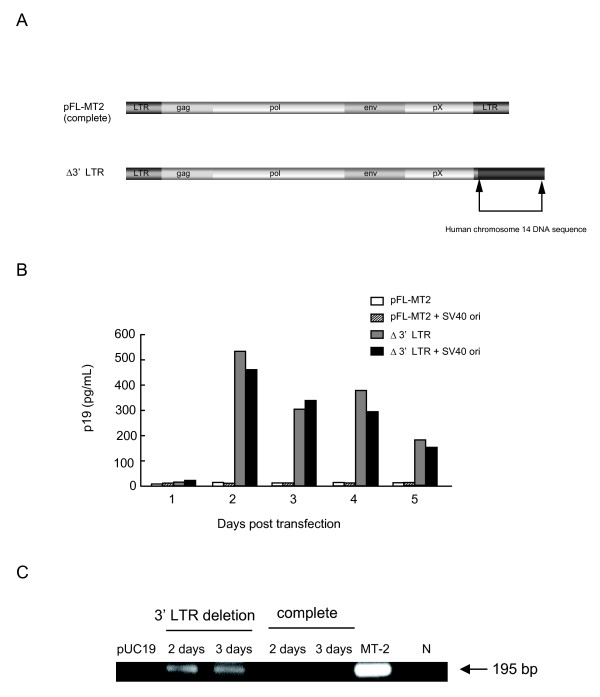
**Increased expression of viral antigens in 293T cells transfected with an HTLV-1 clone with a deleted 3' LTR**. (A) Molecular clones of HTLV-1 constructed by overlapping PCR. The complete genome, pFL-MT2 (upper), and a 3'-LTR-deleted clone in which the 3' LTR was replaced with that of the endogenous human gene (lower). (B) Increased expression of the p19 gag protein was detected by ELISA after transfection with the 3'-LTR-deleted clone. The amount of p19 gag in the concentrated (200-fold) medium from cells transfected with the complete molecular clone, pFL-MT2, pFL-MT2 + SV40 ori, Δ3' LTR, or Δ3' LTR + SV40 ori. (C) RT-PCR detection of the doubly spliced *tax *mRNA in cells transfected with the complete molecular clone or with the Δ3' LTR clone 2-3 days after transfection or with pUC19 two days after transfection. MT-2, total RNA extracted from the HTLV-1-infected cell line MT-2 was used as the positive control; N, total RNA extracted from 293T cells was used as the negative control.

The generation of infectious viruses from cloned proviral DNA is one of the best ways to investigate the biology and pathogenicity of viruses, and to improve methods of disease control. We previously constructed an infectious molecular clone using overlapping polymerase chain reaction (PCR) [13]. 293T cells transfected with this clone alone did not produce virus-like particles, whereas cells transfected with this clone plus a Tax expression vector produced viral-like particles. These cells were then used to produce virus-like particles that were capable of infecting a human T-cell line. In constructing the HTLV-1 provirus by overlapping PCR, we sometimes isolated an incomplete provirus with a deletion in the 3' LTR. To construct the full-length clone, four fragments were constructed by PCR: 1.4-kb 5LTR-gag(-), 3.9-kb gag(+)-pol(-), 2.7-kb pol(+)-SK44, and 1.7-kb SK43-3LTR. The full-length HTLV-1 DNA (9 kb) was synthesized from these four DNA fragments using overlapping PCR. It was sometimes found that the 1.7-kb SK43-3LTR fragment was replaced with the human chromosome 14 DNA sequence during the overlapping PCR process. Therefore, a 3'-LTR-deleted HTLV-1 molecular clone was constructed (Figure 1A). The constructed full-length (pFL-MT2) and 3'-LTR-deleted clones were used to transfect the human epithelial 293T cell line. Transfections of 293T cells were performed as described previously [13]. Viral antigens were detected in the supernatants of cells transfected with these clones using an enzyme-linked immunosorbent assay (ELISA) [13]. Surprisingly, the 3'-LTR-deleted clone produced viral antigen when the clone alone was transfected into 293T cells, whereas 293T cells transfected with the complete HTLV-1 proviral DNA alone did not produce viral antigen (Figure 1B). To increase the plasmid replication in transfected cells, the SV40 origin of replication (ori) was added to these clones but the viral antigen expression levels did not increase in all the clones. Next, *tax *gene expression was confirmed in the cells transfected with the clones using reverse transcription (RT)-PCR [14]. 293T cells transfected with the complete proviral DNA did not express the *tax *gene, whereas 293T cells transfected with the 3'-LTR-deleted clone expressed the *tax *gene (Figure 1C).

**Figure 2:**
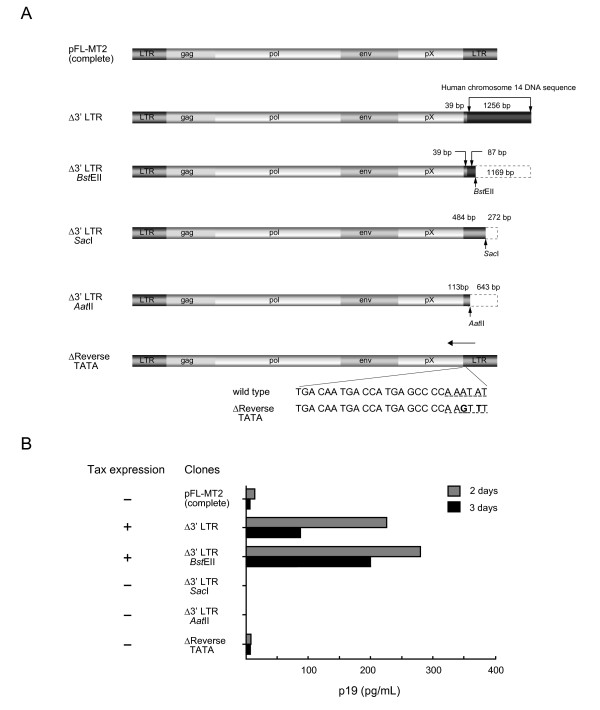
**Various molecular clones with deletions of the 3' LTR of different lengths and the corresponding viral expression patterns**. (A) Diagram of the molecular clones showing the deletion series of the 3' LTR and the deletion of the reverse TATA box of the 3' LTR. (B) The amount of p19 gag in the pooled concentrated (100-fold) medium from 293T cells transfected with the complete molecular clone pFL-MT2 or with clones containing deletions of the 3' LTR of different lengths and the deleted reverse TATA box 2-3 days after transfection. The results of RT-PCR detection of *tax *mRNA in cells transfected with the various clones are shown on the left side.

Next, to confirm the region of the 3' LTR responsible for the increase in viral replication, various clones were constructed with different lengths of the 3' LTR (Figure 2A). A *Bst*EII site was found in the replacement sequence (human chromosome 14 DNA; 1256 bp) of the Δ3' LTR, and removed 1169 bp from the Δ3' LTR. The reconstructed clone had 39 bp of the 3' LTR and 87 bp of human chromosome 14 DNA, and was designated Δ3' LTR *Bst*EII. Δ3' LTR *Sac*I was constructed by removing a 272-bp fragment downstream from the *Sac*I site of the 3' LTR in the complete molecular clone pFL-MT2. Δ3' LTR *Aat*II was constructed by removing a 643-bp fragment downstream from the *Aat*II site of the 3' LTR in the complete molecular clone pFL-MT2 and contained 113 bp of the 3' LTR. The complete HTLV-1 provirus has the same LTR at its 5' and 3' ends. The HTLV-1 LTR has a reverse-direction TATA box [15]. Therefore, to investigate whether the transcripts derived from the reverse direction inhibit HTLV-1 replication, a Δreverse TATA mutant, was constructed by site-specific PCR mutagenesis (GeneTailor™, Invitrogen, Carlsbad, CA, USA), and contained a deletion of the reverse TATA sequence, but with no change in the amino acid sequence of Tax.

These constructed clones were used to transfect the human epithelial 293T cell line. RT-PCR of the transfected 293T cells with Δ3' LTR and Δ3' LTR *Bst*EII demonstrated that the cells expressed mRNA sequences corresponding to the *tax *gene. However, the cells transfected with other Δ3' LTR clones, including the complete provirus, did not express the *tax *gene (Figure 2B). The expression of other HTLV-1 genes (*gag*, *pol*, and *env*) was the same as that of the *tax *gene (data not shown). The Δreverse TATA mutant did not produce the viral antigen. Recently, the expression of the HTLV-1 basic leucine zipper factor (HBZ), an antisense mRNA transcribed from the 3' LTR, has been shown to be consistently expressed in adult T-cell leukemia cells. Thus, HBZ may have a functional role in cellular transformation and leukemogenesis [16]. HBZ was first found to inhibit the Tax-mediated transactivation of viral transcription from the 5' LTR by heterodimerizing with Jun and CREB2 [17]. None of the constructed Δ3' LTR clones contained the promoter for the *HBZ *gene located in U5 and only the part R region of the 3' LTR [18]. No *HBZ *gene transcript was detected by RT-PCR in cells transfected with any clone, including those transfected with the complete provirus clone.

These results suggest that the sequence between 40 nucleotides (nt) and 113 nt at the *Aat*II site downstream from the beginning of the 3' LTR, which constitutes the C-terminal portion of the *tax *downstream sequence, might be involved in the inhibition of the replication of HTLV-1 genes in infectious molecular clones. It is suggested that the complete infectious molecular clone could not produce the viral antigen because the expression of Tax was low, and there might exist a binding site for cellular factors that inhibit the expression of the *tax *gene between 40 nt and 113 nt at the *Aat*II site downstream from the beginning of the 3' LTR. Recently, Fryrear et al. reported that a Tax mutant (353 amino acids), in which the C-terminal portion (amino acids 323-353) was deleted, displayed higher transcriptional activity than that of the wild-type protein [19]. There is a PDZ-protein-binding motif at this site, which interacts with several PDZ proteins, such as DLG1, the precursor of interleukin 16, and MAGI3 [20-25]. Ishioka et al. reported that inactivation of DLG1 augments the Tax-mediated transformation of cells. This finding suggests that DLG1 regulates the function of Tax through its PDZ-binding motif [26]. HTLV-1-infected cells in the peripheral blood rarely express viral genes in HTLV-1-infected individuals [16,27]. This might be caused by cellular factors, such as PDZ proteins, inhibiting Tax expression by binding to the PDZ-protein-binding motif in the C-terminal portion of the *tax *gene.

## Competing interests

The author declares that they have no competing interests.
